# Novel models by machine learning to predict the risk of cardiac disease-specific death in young patients with breast cancer

**DOI:** 10.1007/s12672-024-01676-9

**Published:** 2024-12-18

**Authors:** Yi Li, Handong Li, Xuan Ye, Zhigang Zhu, Yixuan Qiu

**Affiliations:** 1 Department of Geriatrics, Hematology and Oncology Ward, The Second Affiliated Hospital, School of Medicine, South China University of Technology, No.1 Panfu Road, Guangzhou, China; 2 Shuguang Hospital Affiliated to Shanghai University of Traditional Chinese Medicine, Shanghai, China; 3 Department of Breast and Thyroid Surgery, Guangzhou Women and Children’s Medical Center, Guangzhou Medical University, Guangdong Provincial Clinical Research Center for Child Health, Guangzhou, China; 4 Department of Gastroenterology, Guangdong Provincial People’s Hospital (Guangdong Academy of Medical Sciences), Southern Medical University, No. 106 Zhongshan Second Road, Guangzhou, China

**Keywords:** Breast cancer, Cardiac disease, Machine learning, SEER

## Abstract

**Background:**

With the tremendous leap of various adjuvant therapies, breast cancer (BC)-related deaths have decreased significantly. Increasing attention was focused on the effect of cardiac disease on BC survivors, while limited existing population-based studies lay emphasis on the young age population.

**Method:**

Data of BC patients aged less than 50 years was collected from the SEER database. A competing risk model was introduced to analyze the effects of clinicopathology variables on the cardiac disease-specific death (CDSD) risks of these patients. Further, an XGBoost prediction model was constructed to predict the risk of CDSD. Prediction performance was assessed using the receiver operating characteristic (ROC) analysis, area under the POC curve (AUC) values, calibration curves, decision curves, and confusion matrix, and SHapley Additive exPlanations (SHAP) were used to interpret the models.

**Results:**

Our competing risk analysis proved that young BC patients with older age, low household income, non-metropolitan residential environment, black race, unmarried status, HR + subtype, higher T stage (T2-4), receiving chemotherapy, and non-surgery are under higher risk of CDSD. Further, five machine learning models were constructed to predict the CDSD risks of young BC patients, among which the XGBoost models showed the highest AUC value (train set: AUC = 0.846; test set: AUC = 0.836). The confusion matrix of the XGBoost model demonstrated that the sensitivity, specificity, and correction were 0.81, 0.94, and 0.94 for the train set, and 0.82, 0.95, and 0.96 for the test set, respectively. The SHAP graph indicated that median household income, marital status, race, and age at diagnosis were the top four strongest predictors.

**Conclusion:**

Independent CDSD risk factors for young BC patients were identified, and machine-learning prognostic models were constructed to predict their CDSD risks. Our validation results indicated that the predicted probability of our XGBoost model agrees well with the actual CDSD risks, and it can help recognize high-risk populations and therefore develop effective cardioprotection strategies. Hopefully, our findings can support the growth of the new field of cardio-oncology.

## Introduction

Breast cancer (BC) has developed as the top diagnosed cancer globally [1], and in America alone, there are over 260,000 new diagnoses every year [2]. With the tremendous leap of various adjuvant therapies including endocrine therapy, targeted therapy, immune checkpoint inhibitors (anti-programmed cell death protein-1 therapy, anti-PD1 therapy), platinum-based chemotherapeutic agents, the risk of BC-related deaths of patients has been greatly reduced. Increased BC morbidity alongside early diagnosis and great improvements in cancer treatments [3], combined to lead to a larger survivor population, which means a larger population with increased risk of other comorbidities, especially, cardiac disease. Compared with healthy controls, patients with BC are more likely to develop cardiac disease, and those who developed cardiac disease have a significantly bleak prognosis [4, 5]. In a study based on a large cohort population, it was discovered that the BC therapy-related incidence of major cardiac events was 4.1% at 5 years [6]. For patients with pre-existing cardiovascular risk factors, cardiac disease attributed to about 16.3% of deaths among all BC patients, exceeding the mortality due to cancer itself at 10-year follow-up [5]. In general, BC survivors who develop cardiac disease have a 3.8-fold higher all-cause mortality than those who do not [4]. Therefore, more focus should be paid to cardiac disease-specific death (CDSD) for patients with BC.

Normally, older age was considered and proved to be related to a higher rate of cardiac disease [6]. Therefore, enormous studies put emphasis on cardiac events in the old age cohort. CDSD risk in young women with BC remains a giant challenge, not only because of its rarity but also because of the lack of modifiable risk factors and the shortage of screening for young patients. Normally, young patients are prone to receive more intensive treatment compared with older women, and more aggressive therapies equals higher toxicity. The incidence of cancer treatment-associated cardiac dysfunction is 9–26% during or soon after the completion of cancer therapy for BC patients who received doxorubicin, 13–17% for those who received trastuzumab, and 27–34% for those who received combination therapies [7–11]. Despite facing high risk of cardiac disease development, uncertainties persist about the risk of CDSD among young BC patients on specific subgroups such as cancer stage, treatment, race, etc.

This study aims to predict the risk of CDSD in young BC patients after various treatment by means of machine learning models [12, 13], thus providing a wake-up call for clinical care and monitoring of this group of patients. Hopefully, these findings will serve as a credible epidemiological foundation for tailored management of young BC patients and assist healthcare systems in reducing the risk of CDSD burden among these patients.

## Materials and methods

### Data source and study design

Data about BC patients analyzed in this study were collected from the SEER database [SEER 17 Regs study data, (changes 2000–2021); version 8.4.3], which is openly accessible. Inclusion criteria: (1) female patients with BC; (2) all patients had histopathological and morphological evidence according to the International Classification of Cancer Diseases Edition III (ICD-O-3); (3) patients aged less than 50 years. Exclusion criteria: (1) not primary BC according to international rules; (2) patients with unknown survival time. The patients’ death, loss to follow-up, or December 31, 2021 is the endpoint of the follow-up.

### Model construction

Feature selection: univariate fine-gray competition risk analyses were performed on clinical characteristics. Characteristics that were statistically significant in the univariate Fine-gray competition risk, including age at diagnosis, median household income, rural–urban continuum code, race, hormone receptor (HR) subtype, marital status, grade, T stage, N stage, M stage, radiotherapy, chemotherapy, surgery, were incorporated into machine learning models for CDSD risk prediction in young BC patients. A response variable was collected for causes of death information before running the training program, in which “1” = death from cardiac disease and “0” = alive or death from other causes. Patients were randomized into train data and test data in a 7:3 ratio. We also compared the area under the curve (AUC value) of logistic regression (LR), support vector machine (SVM), random forest (RF), decision tree (Iterative Dichotomiser 3, ID3), and Extreme Gradient Boosting (XGBoost) models on train and test data. LR is a classification algorithm also known as logarithmic dominance regression, it is an interpretable algorithm and a hallmark of classical predictive modeling. SVM is a binary classification model that aims to find a hyperplane to segment the samples and the principle of segmentation is interval maximisation. In ID3, nodes represent input factors and leaves denote decision outcomes. RF can increase the uncertainty of the model by randomly selecting features and randomly dividing the dataset to reduce the overfitting, therefore it is an integrated learning method based on multiple decision trees. Finally, XGBoost is an algorithm based on Gradient Boosting, which constructs multiple decision trees iteratively, and gradually optimizes the loss function through the gradient descent method. It adds a regularisation term to the loss function to gradually optimize the predictive performance of the model.

Further, receiver operating characteristic (ROC) analysis, AUC values, calibration curves, decision curves, and confusion matrix were employed to evaluate our model. Sensitivity, specificity, and correction are the primary assessment parameters in the confusion matrix. Our XGBoost model was visualised using SHapley Additive exPlanations (SHAP) values. SHAP is an ex-post model interpretation method, and its core idea is to compute the marginal contribution of features to the model output, and then interpret the "black box model" both globally and locally.

### Statistical analysis

Categorical variables were expressed as frequencies and percentages, and continuous variables were expressed as means and standard deviations (M ± SD). The between-group comparison of categorical data was achieved using the χ^2^ test or Fisher’s exact test. To explore the association between various clinicopathology characteristics and the risk of CDSD in patients, the univariate Fine-gray competition model was introduced. To assess patients’ risk of CDSD and to identify independent risk factors, multivariate Fine-gray analyses were performed on variables that were statistically different in univariate analysis. In the competition risk analysis, deaths from CDSD were considered as target events, and deaths from other causes were considered as competition risk events. All statistical calculations were performed using the R programming language (version 4.0.2). Statistical significance was defined as a two-sided tail value of less than 0.05.

## Results

### Clinical characteristics of young BC patients

A total of 25,3362 patients aged younger than 50 years were incorporated in this study, among which, 209,067 of them survived, 1275 of them died from CDSD, and 43,320 of them died from other cause. The detailed clinicopathological information of young patients with BC are shown in Table 1 and generalized below. The median age at diagnosis of patients who experienced CDSD was 44.29 ± 4.47 years, significantly higher than those who were alive (42.75 ± 5.35 years) and those who died due to other causes (42.01 ± 5.77 years). Most of the patients who suffered CDSD were white (801 cases, 62.8%), unmarried (657 cases, 51.5%), HR-positive (HR +; 195cases, 15.3%), grade III/IV (591 cases, 46.4%), IDC histological type (963 cases, 75.5%), and lived in the counties in metropolitan areas with over1 million population (658 cases, 51.7%),

**Table 1:** Baseline characteristics of younger patients with breast cancer

	Alive	Cardiac Diease Specific Death	Other Causes of Death	*P* Value
	N(%) / Mean(SD)	N(%) / Mean(SD)	N(%) / Mean(SD)	
Age at diagnosis	42.75 (5.35)	44.29 (4.47)	42.01 (5.77)	***
Median Household Income				***
Less than 50,000	9017 (4.3%)	137 (10.7%)	2621 (6.1%)	
50,000–59,999	14,086 (6.7%)	150 (11.8%)	3816 (8.8%)	
60,000–69,999	27,929 (13.4%)	241 (18.9%)	6670 (15.4%)	
70,000–79,999	50,026 (23.9%)	309 (24.2%)	12,365 (28.6%)	
80,000–89,999	34,679 (16.6%)	163 (12.8%)	6332 (14.6%)	
90,000–99,999	30,531 (14.6%)	120 (9.4%)	5209 (12.0%)	
100,000–109,999	19,447 (9.3%)	97 (7.6%)	3168 (7.3%)	
110,000–119,999	11,574 (5.5%)	35 (2.7%)	1719 (4.0%)	
More than 119,999	11,741 (5.6%)	23 (1.8%)	1404 (3.2%)	
Unknown	37 (< 0.1%)	0 (0%)	16 (< 0.1%)	
Rural–Urban Continuum Code				***
Counties in metropolitan areas ge 1 million pop	135,787 (64.9%)	658 (51.6%)	26,795 (61.8%)	
Counties in metropolitan areas of 250,000 to 1 million pop	42,201 (20.2%)	265 (20.8%)	8474 (19.6%)	
Counties in metropolitan areas of lt 250 thousand pop	13,834 (6.6%)	136 (10.7%)	3409 (7.9%)	
Nonmetropolitan counties	16,920 (8.1%)	213 (16.7%)	4550 (10.5%)	
Unknown	325 (0.2%)	3 (0.2%)	92 (0.2%)	
Race				***
White	156,072 (74.7%)	801 (62.8%)	30,389 (70.2%)	
Black	23,019 (11.0%)	401 (31.5%)	8701 (20.1%)	
Other	27,905 (13.3%)	72 (5.6%)	4134 (9.5%)	
Unknown	2071 (1.0%)	1 (0.1%)	96 (0.2%)	
Hormone receptor				***
Negative	38,841 (18.6%)	323 (25.3%)	12,866 (29.7%)	
Positive	158,090 (75.6%)	781 (61.3%)	25,584 (59.1%)	
Unknown	12,136 (5.8%)	171 (13.4%)	4870 (11.2%)	
Human epidermal growth factor receptor-2				***
Negative	93,365 (44.7%)	195 (15.3%)	10,684 (24.7%)	
Positive	24,402 (11.7%)	51 (4.0%)	2533 (5.8%)	
Unknown	91,300 (43.7%)	1029 (80.7%)	30,103 (69.5%)	
Marital status				***
Unmarried	65,400 (31.3%)	657 (51.5%)	17,667 (40.8%)	
Married	135,147 (64.6%)	562 (44.1%)	23,693 (54.7%)	
Unknown	8520 (4.1%)	56 (4.4%)	1960 (4.5%)	
Grade				***
I	33,156 (15.9%)	141 (11.1%)	2289 (5.3%)	
II	80,308 (38.4%)	397 (31.1%)	12,356 (28.5%)	
III/IV	80,263 (38.4%)	591 (46.4%)	23,299 (53.8%)	
Unknown	15,340 (7.3%)	146 (11.5%)	5376 (12.4%)	
Histological type				***
IDC	164,641 (78.8%)	963 (75.5%)	32,407 (74.8%)	
ILC	12,447 (6.0%)	58 (4.5%)	2361 (5.5%)	
Other	31,979 (15.3%)	254 (19.9%)	8552 (19.7%)	
T stage				***
T1	110,759 (53.0%)	514 (40.3%)	11,002 (25.4%)	
T2	70,491 (33.7%)	484 (38.0%)	15,900 (36.7%)	
T3	14,457 (6.9%)	110 (8.6%)	5886 (13.6%)	
T4	4469 (2.1%)	84 (6.6%)	5182 (12.0%)	
Unknown	8891 (4.3%)	83 (6.5%)	5350 (12.3%)	
N stage				***
N0	127,952 (61.2%)	653 (51.2%)	13,200 (30.5%)	
N1	59,579 (28.5%)	386 (30.3%)	14,730 (34.0%)	
N2	11,595 (5.5%)	109 (8.5%)	6133 (14.2%)	
N3	5762 (2.8%)	67 (5.3%)	6130 (14.2%)	
Unknown	4179 (2.0%)	60 (4.7%)	3127 (7.2%)	
M stage				***
M0	202,811 (97.0%)	1169 (91.7%)	34,464 (79.6%)	
M1	3946 (1.9%)	75 (5.9%)	7475 (17.3%)	
Unknown	2310 (1.1%)	31 (2.4%)	1381 (3.2%)	
Radiotherapy				***
No	101,585 (48.6%)	698 (54.7%)	22,718 (52.4%)	
Yes	107,482 (51.4%)	577 (45.3%)	20,602 (47.6%)	
Chemotherapy				***
No	81,276 (38.9%)	446 (35.0%)	10,962 (25.3%)	
Yes	127,791 (61.1%)	829 (65.0%)	32,358 (74.7%)	
Surgery				***
No	9257 (4.4%)	119 (9.3%)	7946 (18.3%)	
Yes	199,436 (95.4%)	1149 (90.2%)	35,154 (81.2%)	
Unknown	374 (0.2%)	7 (0.5%)	220 (0.5%)	

*P < 0.05, **P < 0.01, ***P < 0.001

As for median household income, the lower the household income, the higher the risk of patients to experience CDSD. A similar trend was found in rural–urban area where patients living, those who lived in metropolitan areas with a larger population basis had a lower risk of CDSD, while those who came from nonmetropolitan counties had the greatest risk of CDSD. Moreover, among all races and marital statuses, black people and unmarried patients had the highest chance of dying from cardiac disease.

### Competition risk analysis of cardiac disease-specific death

In this study, univariate and multivariate Fine-Gray competition risk analysis was performed to analyze the CDSD for the young BC patients (Table 2). The results of univariate Fine-Gray analysis showed that age at diagnosis, median household income, rural–urban residential environment, HR subtype, grade, TNM stage, radiotherapy, chemotherapy, and surgery significantly affected the CDSD (*P* < 0.05). However, human epidermal growth factor receptor 2 (HER2) status and histological type were not associated with CDSD (*P* > 0.05). The factors that were statistically significant in the univariate analysis (*P* < 0.05) were added to the multivariate Fine-Gray competition risk model. Compared with the findings from the baseline characteristics, it was proved that age was still an independent risk factor for the CDSD occurrence of young BC patients (HR = 1.077; 95% CI = 1.062–1.093, *P* < 0.001). The black population (vs white: HR = 2.391, 95% CI = 2.046–2.794, *P* < 0.001) had the highest CDSD risk among patients of all races. Patients with higher household income (HR = 0.927; 95% CI = 0.887–0.968, *P* < 0.001) and married marital status (vs unmarried: HR = 0.523, 95% CI = 0.457–0.598, *P* < 0.001) always had lower CDSD risk, while nonmetropolitan residential environment (vs counties in metropolitan areas over 1 million population HR = 1.785; 95% CI = 1.428–2.232, *P* < 0.001) and HR + subtype (vs HR-negative: HR = 1.428; 95% CI = 1.104–1.675, *P* < 0.005) always correlated with higher CDSD risk. Higher T stage were correlated with higher CDSD risk, obviously, young BC patients with T2 (vs T1: HR = 1.372, 95% CI = 1.175–1.610, *P* < 0.001), T3 (vs T1: HR = 1.452, 95% CI = 1.130–1.866, *P* < 0.01), and T4 (vs T1: HR = 1.363, 95% CI = 1.101–1.562, *P* < 0.05) had a significant higher CDSD risk than those in T1 stage. Surgery (vs no surgery: HR = 0.840, 95% CI = 0.735–0.959, *P* < 0.05) can help induce the risk of CDSD for young BC patients. However, chemotherapy (vs no chemotherapy: HR = 1.322, 95% CI = 1.102–1.689, *P* < 0.05) will significantly increase the risk of developing CDSD. Moreover, N stage, M stage, tumor grade, and radiotherapy are not independent risk factors for CDSD of young BC patients.

**Table 2:** Univariate and multivariate Fine-Gray competition risk analysis of characteristics

	Heart Diease Specific Death					
	Univariate analysis			Multivariate analysis		
	HR	95%CI	*P* Value	HR	95%CI	*P* Value
Age at diagnosis	1.070	1.05–1.08	***	1.077	1.062–1.093	***
Median Household Income	0.815	0.789–0.842	***	0.927	0.887–0.968	***
Rural–Urban Continuum Code						
Counties in metropolitan areas ge 1 million pop	Reference			Reference		
Counties in metropolitan areas of 250,000 to 1 million pop	1.280	1.110–1.480	***	1.272	1.072–1.508	**
Counties in metropolitan areas of lt 250 thousand pop	1.780	1.480–2.140	***	1.472	1.158–1.870	**
Nonmetropolitan counties	2.220	1.900–2.590	***	1.785	1.428–2.232	***
Race						
White	Reference			Reference		
Black	3.003	2.663–3.386	***	2.391	2.046–2.794	***
Other	0.616	0.484–0.784	***	0.699	0.523–0.936	*
Hormone Receptor						
Negative	Reference			Reference		
Positive	1.547	1.256–1.851	***	1.428	1.104–1.675	*
HER2						
Negative	Reference					
Positive	1.010	0.745–1.380	0.930	/	/	/
Marital status						
Unmarried	Reference			Reference		
Married	0.434	0.383–0.491	***	0.523	0.457–0.598	***
Grade						
I	Reference			Reference		
II	1.080	0.890–1.310	0.440	0.991	0.801–1.227	0.940
III/IV	1.340	1.120–1.610	**	1.068	0.851–1.340	0.570
Histological type						
IDC	Reference					
ILC	0.815	0.625–1.060	0.130	/	/	/
Other	1.066	0.929–1.220	0.360	/	/	/
T stage						
T1	Reference			Reference		
T2	1.450	1.280–1.640	***	1.372	1.175–1.601	***
T3	1.500	1.222–1.850	***	1.452	1.130–1.866	**
T4	2.200	1.750–2.780	***	1.363	1.101–1.562	*
N stage						
N0	Reference			Reference		
N1	1.140	1.022–1.290	*	1.069	0.914–1.251	0.400
N2	1.170	0.956–1.440	0.130	1.023	0.799–1.311	0.850
N3	1.170	0.909–1.500	0.230	0.934	0.685–1.273	0.660
M stage						
M0	Reference			Reference		
M1	1.470	1.070–2.010	*	1.138	0.764–1.695	0.520
Radiotherapy						
No	Reference					
Yes	0.794	0.711–0.887	***	0.840	0.743–1.020	0.087
Chemotherapy						
No	Reference					
Yes	1.668	1.339–2.122	***	1.322	1.102–1.689	*
Surgery						
No	Reference					
Yes	0.591	0.490–0.714	***	0.840	0.735–0.959	*

*P < 0.05, **P < 0.01, ***P < 0.001

### Establishment and evaluation of predictive models for estimating the cardiac disease-specific death of young BC patients

**Fig. 1:**
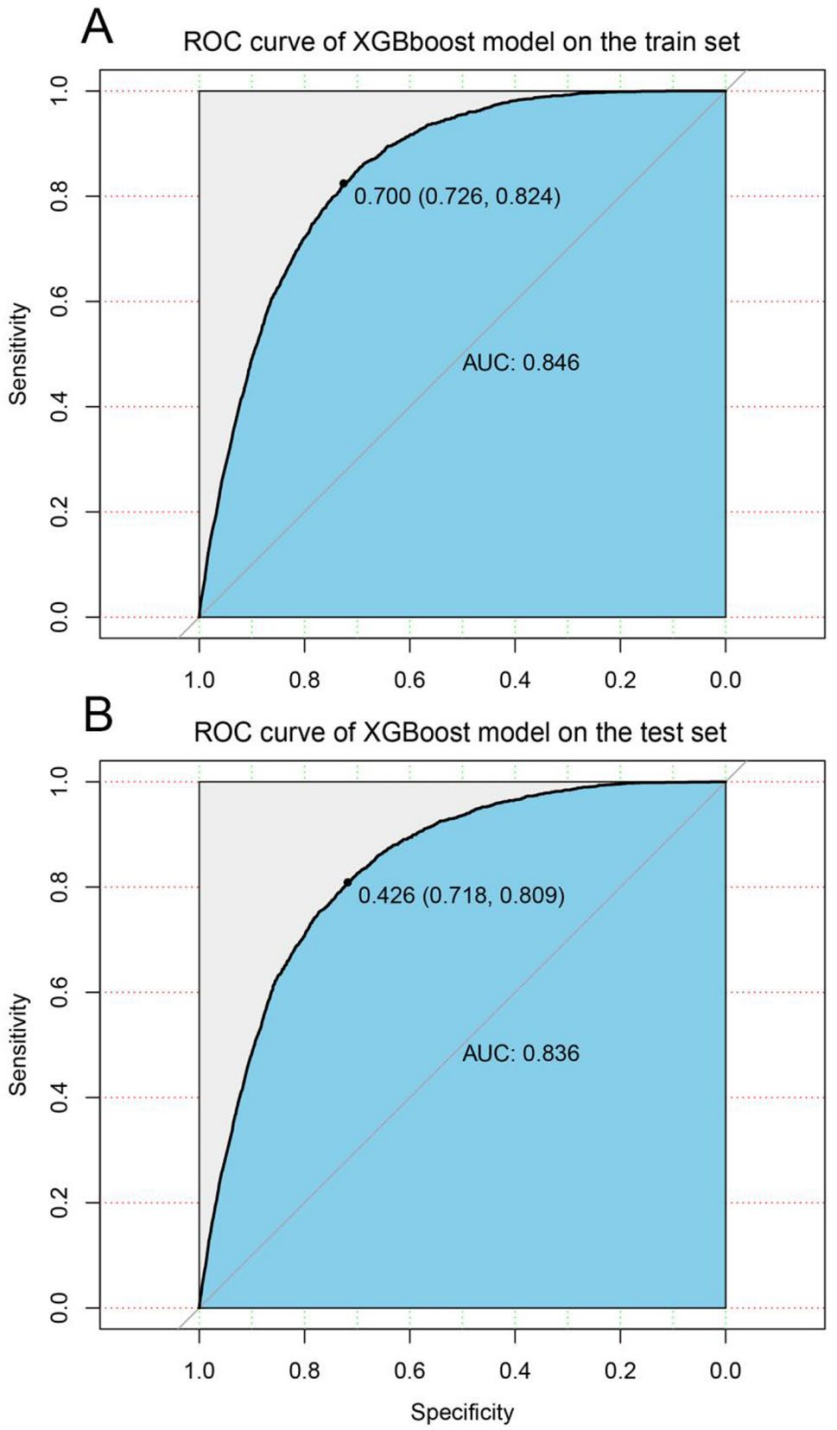
ROC curves of the XGBoost model’s predicted results in the train and test set. **A** ROC curve on the train data; **B** ROC curve on the test data; *ROC* receiver operating characteristic curve, *AUC* area under the curve

To further discriminate the exact patients at high risk of CDSD, novel models based on machine learning were constructed to predict the CDSD risk of young BC patients. The patients were divided into train and test data groups in a 7:3 ratio. To guarantee the stability and reliability of our model, ten-fold cross-validation was performed in the train set for iterative testing and tuning, and therefore generate the optimal model (gamma = 0.1, min_child_weight = 500, scale_pos_weight = 90, subsample = 0.5, max_delta_step = 6, alpha = 2, max_depth = 5, eta = 0.1, nround = 100) (Table 3). To evaluate our model, receiver operator characteristic (ROC) curves were plotted for the train and test set, respectively, and the corresponding area under the ROC curves (AUC) was calculated (Fig. 1). It can be seen that the XGBoost model had an outstanding performance in terms of predicting CDSD risk for young BC patients (train set: AUC = 0.846; test set: AUC = 0.836). To exhibit the performance of our model in a clearer manner, our XGBoost model was compared with several traditional machine learning algorithms, including LR (train set: AUC = 0.755; test set: AUC = 0.746), RF (train set: AUC = 0.820; test set: AUC = 0.803); SVM(train set: AUC = 0.735; test set: AUC = 0.644), ID3 (train set: AUC = 0.799; test set: AUC = 0.785), and our XGBoost model has the highest AUCs value (Table 4).

**Table 3:** Main parameters of the XGBoost model

Parameter	Value
gamma	0.1
min_child_weight	500
scale_pos_weight	90
subsample	0.5
max_delta_step	6
alpha	2
max_depth	5
eta	0.1
nround	100

**Table 4:** Performance of prognostic models built by machine learning algorithms on train and test data (area under the ROC curve)

	Train set	Test set
XGBoost	0.846	0.836
LR	0.755	0.746
RF	0.820	0.803
SVM	0.735	0.644
ID3	0.799	0.785

*XGBoost* extreme gradient boosting, *LR* logistic regression, *RF* random forest, *SVM* support vector machine, *ID3* decision tree

**Fig. 2:**
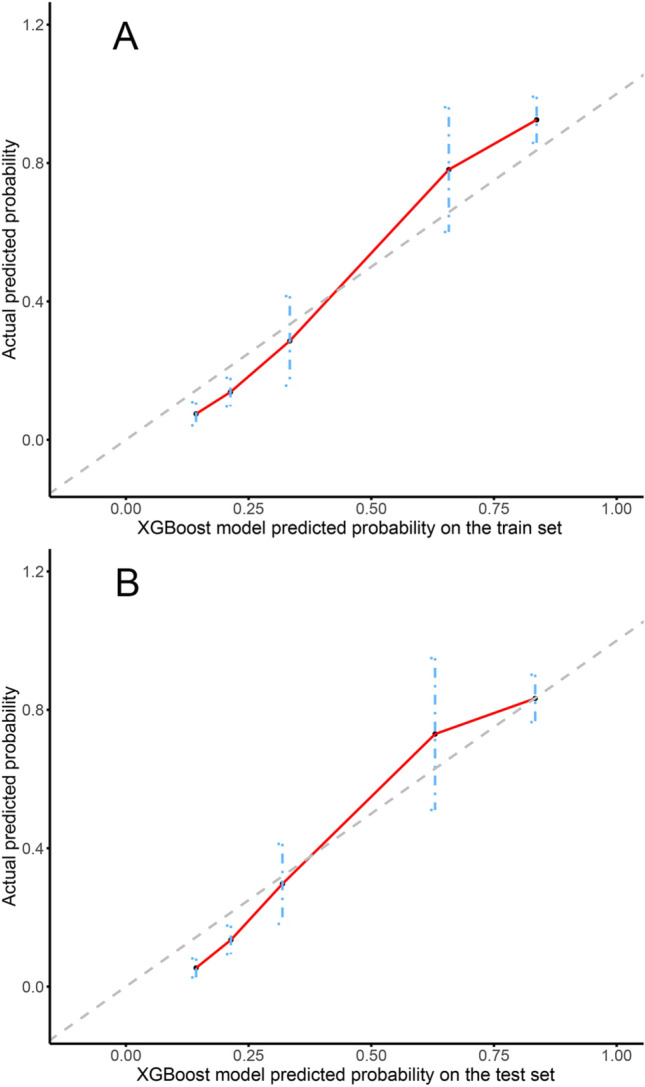
Calibration curves of the XGBoost model’s predicted results in the train and test set. **A** Calibration curve on the train data; **B** Calibration curve on the test data

**Fig. 3:**
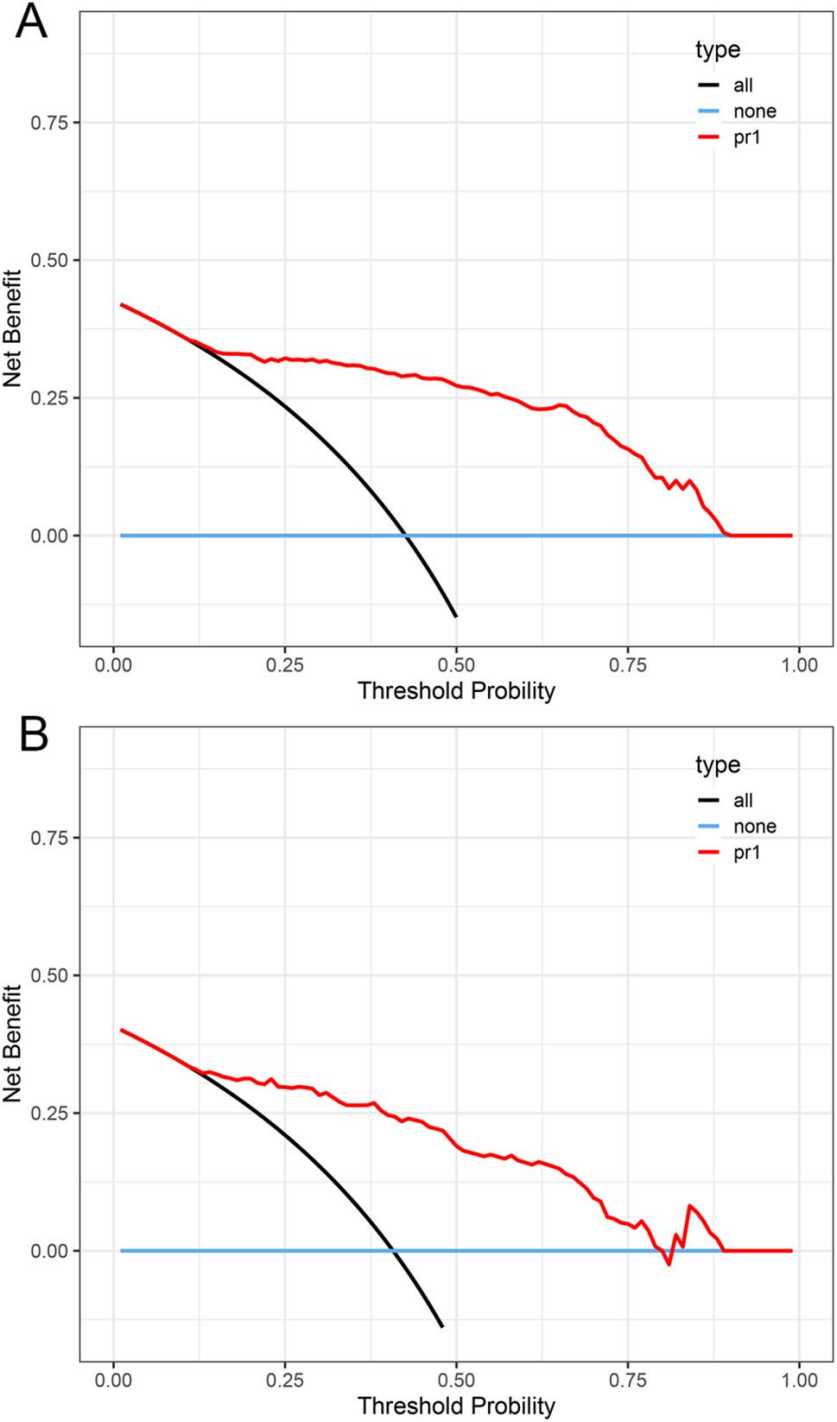
Decision curves of the XGBoost model’s predicted results in the train and test set. **A** Decision curve on the train data; **B** Decision curve on the test data

The closer the calibration curve matches the standard line, the more accurately the dataset’s actual class distribution is predicted by the model. The calibration curve demonstrates that, in both the train set and test set, the predicted probability of our XGBoost model agrees well with the actual risk (Fig. 2). Our model’s predicted value and the actual probability of the result are greatly comparable. Further, decision curve analysis was performed to assess the clinical utility of our model [14]. The decision curves analysis shows a more net benefit than full or no treatment across a threshold probability range in the train (Fig. 3A) and test (Fig. 3B) sets.

**Fig. 4:**
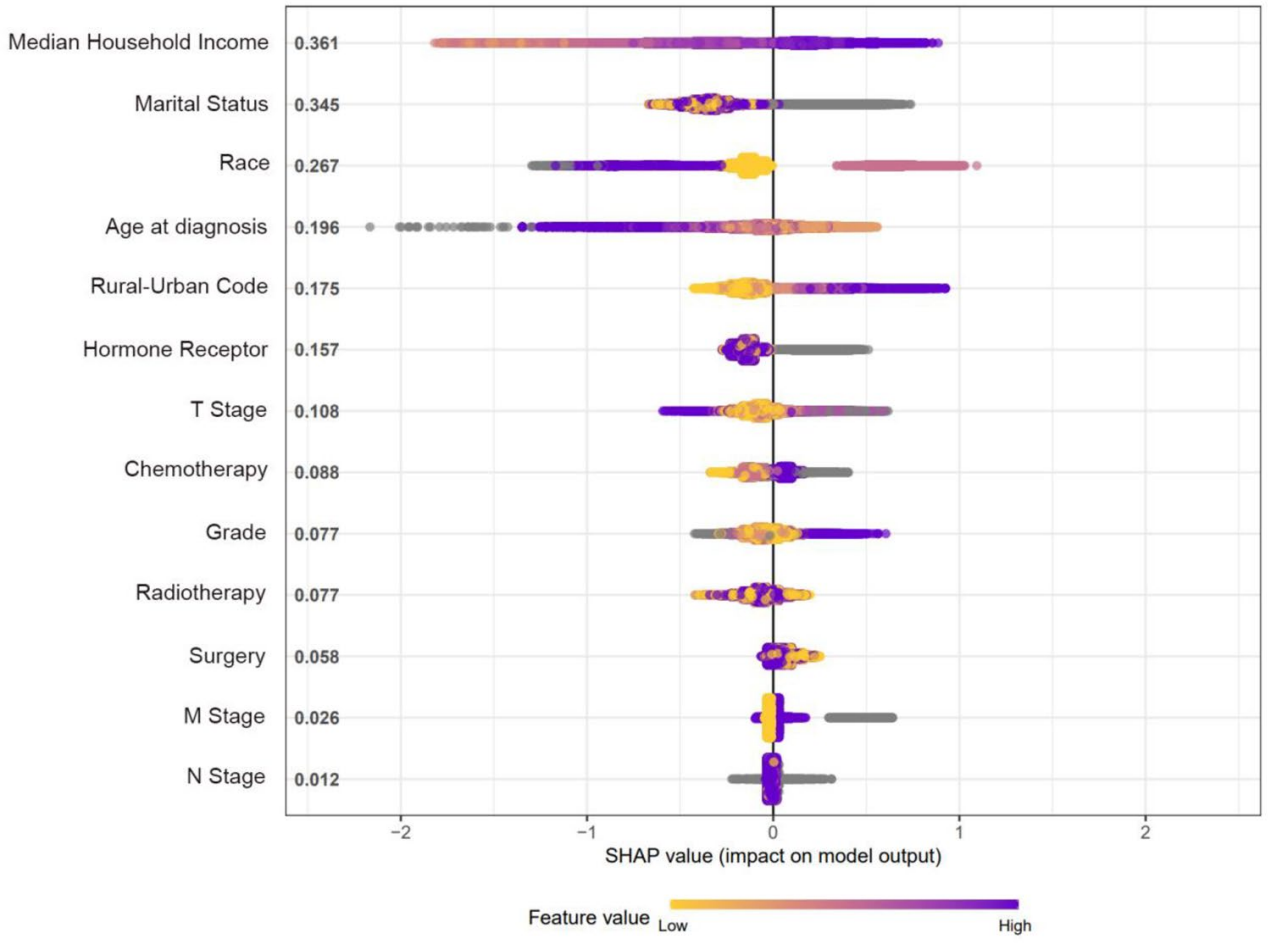
The SHAP value of clinical characteristics in terms of importance in the XGBoost model

The contribution of each variable to the final prediction was illustrated by the SHAP values, which can help clarify and evaluate model predictions for each individual patient. The final SHAP value which corresponded to the predicted score was provided by the combined effect of all factors. The SHAP value for each sample is represented by each point on the graph; the point with a color closer to purple equals to a higher value, while that with a color closer to yellow reflects a lower value. The more dispersed the points in the graph, the stronger effect of the variable has on the model. The results showed that median household income, marital status, race, and age at diagnosis were the top four strongest predictors (Fig. 4).

**Fig. 5:**
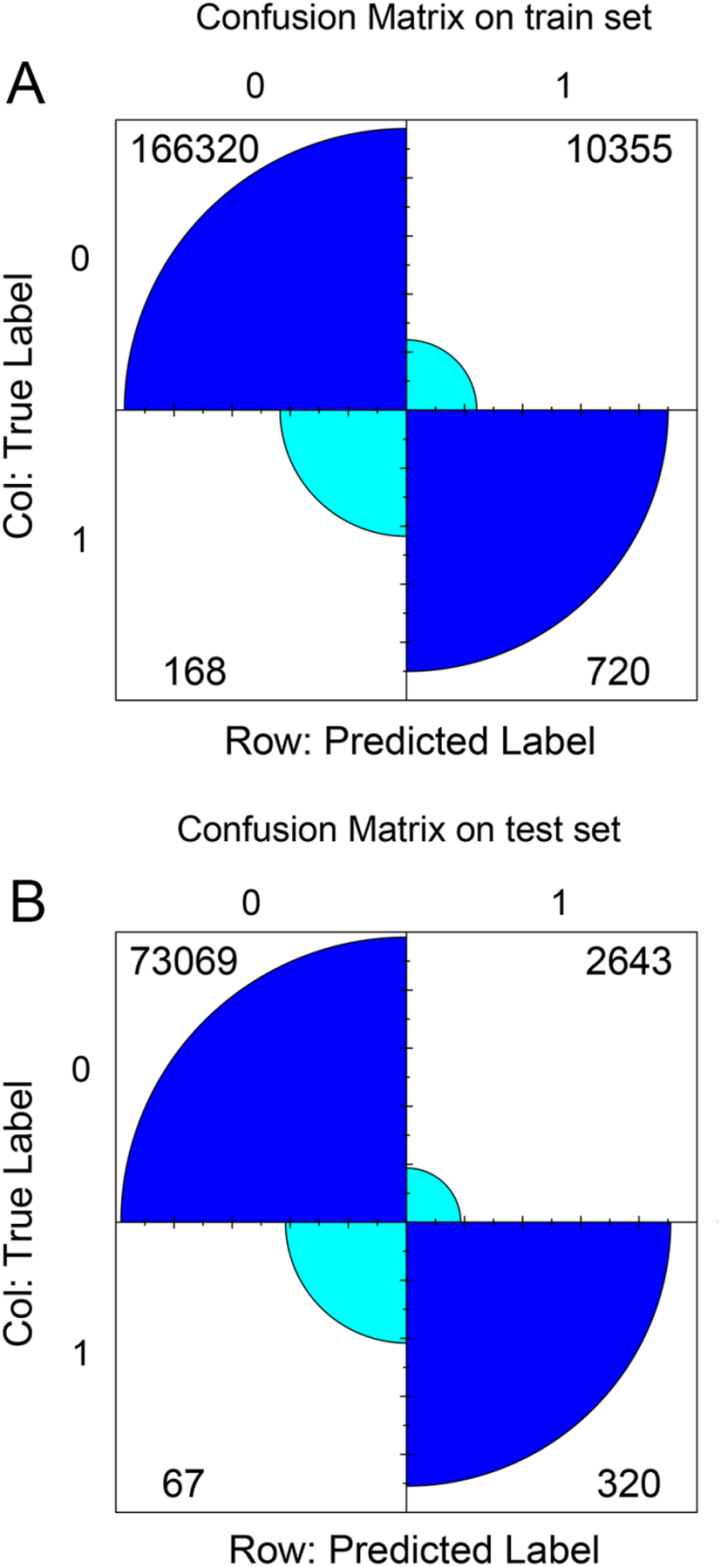
Confusion matrix of the XGBoost model’s predicted results in the train and test set. **A** Confusion matrix on the train set; **B** Confusion matrix on the test set

From the confusion matrix of the XGBoost model (Fig. 5), it was calculated that the sensitivity, specificity, and correction were 0.81, 0.94, and 0.94 for the train set, and 0.82, 0.95, and 0.96 for the test set, respectively. The results proved that our model showed extraordinary performance while predicting the CDSD risk for young BC patients.

## Discussion

BC patients have a higher risk of developing cardiac disease compared to non-cancer controls [15]. A previous population-based study with over 40 years of follow-up assessed the cardiac disease death risk for patients from 21 cancer sites. It discovered cardiac disease death risk as the most significant competing risk clinically for most non-metastatic cancers, and in BC, CDSD had surpassed primary tumor gradually as the primary cause of death [16]. Once the cardiac events occur, the patients always face significantly worse overall survival outcomes, and in certain BC populations, CDSD exceeds cancer death rates. Therefore, it is imperative to recognize high-risk populations and develop effective cardioprotection strategies.

When it comes to cardiac diseases, the old age population always comes to mind. Therefore, most previous studies put emphasis on this population and neglected the young age patients. A number of researches have documented that, young women have a larger chance of developing more aggressive subtypes of BC with poor prognostic features, and also present with more advanced disease stages [17, 18]. Young BC patients conventionally receive more intensive therapies compared with old women, but they still suffer from a higher risk of BC recurrence and death [19]. The longer survival time of young patients combined with higher toxicity caused by more aggressive therapies, highlights the non-negligible risk of CDSD for them. This study aims to assess the risk of CDSD for young BC patients from various specific clinicopathological and psychosocial subgroups.

Our baseline clinical characteristic analysis and competing risk analysis combined to prove that young BC patients with older age, low household income, non-metropolitan residential environment, black race, unmarried status, HR + subtype, higher T stage (T2-4), receiving chemotherapy, and non-surgery are under higher risk of CDSD. The racial disparity in the risk of CDSD might be because, hypertension rates among black patients rank the highest in the world according to estimates, and hyperaldosteronism is significantly correlated with cardiovascular risk [20, 21]. Household income, which reflects economic stability [22], was found to be the strongest risk factor for CDSD, this supports the findings proving the correlation between atherogenesis and a proinflammatory state and low socioeconomic status [23, 24]. These social determinants including racial disparities, marriage status, household income, and residential environment, combined to urge us to strengthen the management of rural and low-income families and highlight the indispensable role of family support.

The explanation behind the increased risk of CDSD may be multifactorial. For the first thing, smoking, hypertension, diabetes, nutritional factors, micronutrient deficiencies, and other common risk factors for cardiac disease and cancer may exacerbate the CDSD risk [25–27]. The prolonged survival times provided by both the tremendous leap of various BC therapies and the young age (< 50 years) of young BC patients combined to contribute to a higher chance of being exposed to the above-mentioned risk factors and consequently higher CDSD risk. This can also explain the higher CDSD risk of patients with HR + subtype due to their better prognosis and longer survival. Secondly, we must not neglect the cardiotoxicity of various cancer treatments [28], especially various chemotherapy, such as anthracyclines [29, 30], and HER-2 antagonists (e.g. trastuzumab) ^[31]^. This may explain the higher CDSD risk of BC patients with higher T stage and who did not receive surgery. The patients with advanced stage might lose the chance to perform surgery and therefore are always exposed to longer periods of chemotherapy, which consequently aggravate the cardiotoxicity. Finally, mounting studies have proved that systemic vasculature and heart damage are caused by the tumor itself. It was discovered that neutrophil extracellular trap (that is cancer-induced inflammation) may accumulate in vasculature and heart, leading to cardiovascular dysfunction [32, 33].

Although for patients with BC, tumor management should be the top priority, our data highlighted the importance of competing risks. Besides tumors, BC patients also have a great chance to die from other causes, chief among which is cardiovascular disease. Therefore, to better discriminate the patients at high risk of CDSD, we constructed a robust XGBoost model for young BC patients. In general, our XGBoost model demonstrated good performance, indicating the high clinical value of our model. Moreover, our findings highlight the necessity to take into account competing risks, particularly in the development of risk assessment tools. Moreover, our affirmation of the indispensable role of cardiac disease as a competing risk among young BC patients with supports the growth of the new field of cardio-oncology. The limitations of this study are its retrospective nature, and the deficiencies inherent in the SEER database.

## Conclusion

We identified independent CDSD risk factors for young BC patients and constructed machine-learning prognostic models to predict their CDSD risks. Our validation results indicate that the predicted probability of our XGBoost model agrees well with the actual CDSD risks, and it can help recognize high-risk populations and develop effective cardioprotection strategies. Hopefully, our findings can support the growth of the new field of cardio-oncology.

## Data Availability

All data here are publicly available in the SEER database: https://seer.cancer.gov/.
